# Enantioselective Metabolism of Quizalofop-Ethyl in Rat

**DOI:** 10.1371/journal.pone.0101052

**Published:** 2014-06-25

**Authors:** Yiran Liang, Peng Wang, Donghui Liu, Zhigang Shen, Hui Liu, Zhixin Jia, Zhiqiang Zhou

**Affiliations:** 1 Department of Applied Chemistry, China Agricultural University, Beijing, PR China; 2 Institute of Materia Medica, Chinese Academy of Medical Sciences and Peking Union Medical College, Beijing, PR China; Universidade Federal do Rio de Janeiro, Brazil

## Abstract

The pharmacokinetic and distribution of the enantiomers of quizalofop-ethyl and its metabolite quizalofop-acid were studied in Sprague-Dawley male rats. The two pairs of enantiomers were determined using a validated chiral high-performance liquid chromatography method. Animals were administered quizalofop-ethyl at 10 mg kg^−1^ orally and intravenously. It was found high concentration of quizalofop-acid in the blood and tissues by both intragastric and intravenous administration, and quizalofop-ethyl could not be detected through the whole study which indicated a quick metabolism of quizalofop-ethyl to quizalofop-acid in vivo. In almost all the samples, the concentrations of (+)-quizalofop-acid exceeded those of (−)-quizalofop-acid. Quizalofop-acid could still be detected in the samples even at 120 h except in brain due to the function of blood-brain barrier. Based on a rough calculation, about 8.77% and 2.16% of quizalofop-acid were excreted through urine and feces after intragastric administration. The oral bioavailability of (+)-quizalofop-acid and (−)-quizalofop-acid were 72.8% and 83.6%.

## Introduction

Pesticide is a double edged sword, which plays very important roles in increasing crop production and income, but it also causes some negative effects, such as environmental pollutions [1], [2], homicidal and suicidal accident [3], cancer and other diseases [4]. Among the total amount of pesticide in china, more than 40% of them are chiral [5], and this ratio is increasing as more and more complex structures are being developed. Chiral pesticides are composed of two or multiple enantiomers, which have the same physical, chemical properties and affection in achiral environment. However, for the individual enantiomers can interact enantioselectively with enzymes or biological receptors in organisms [6], the biological and physiological properties of enantiomers are often different [7]. For example, (−)-o,p′-DDT is a more active estrogen-mimic in rat and human than (+)-o,p′-DDT [8]. The (R)-form of dichlorprop is active while the other is totally inactive [9], but its inactive form still has oxidative damage to the non-target organisms [10]. Although the enantioselective ecotoxicities of some chiral pesticides to non-target animals, plants and human cancer cell lines have been reported [7], the different properties of the enantiomers are still poorly understood and many chiral pesticides are still used and regulated as if they were achiral.

**Figure 1:**
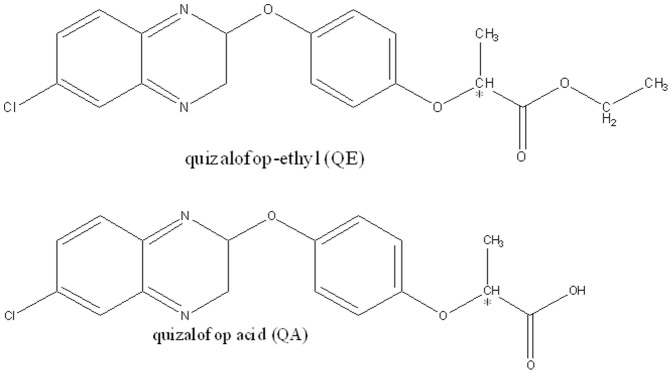
Chemical structures of QE and its primary metabolite QA. Chiral center is denoted by an asterisk (*).

Quizalofop-ethyl, (2RS)-[(2-(4-((6-chloro-2-quinoxalinyl)oxy)phenoxy)-ethyl ester] (QE, Fig. 1) is intensively used to control both annual and perennial grass weeds in broadleaf crops, such as alfalfa, bean, cabbage, canola, carrot, lettuce, potato, soybean, sugar beet, tobacco, tomato and turnip [11]. The half-life (T_1/2_) of quizalofop ethyl on onion was about 0.8 day [12]. QE could be rapidly metabolized to its primary metabolite quizalofop-acid (QA) in soybean, cotton foliage and goat [11], [13]. The study of potential effects of QE on the development of rats has been conducted, and the results showed a significant decrease in the number of fetuses alive and a significant increase in the number of rats with retained placenta [14]. QE exits two enantiomeric forms, the (+)- and (−)-form, but the (+)-form has higher herbicidal activities. For the herbicidal mechanism of QE is inhibiting acetyl CoA carboxylase and (+)-form is a more potent inhibitor against acetyl-CoA in chloroplasts [15]. However, the racemate of QE is still widely used owning to the low cost. The inactive enantiomer just causes environmental problems and may have influences on non-target organisms after their use on crops.

A chiral HPLC and a LC-MS/MS method were set up for the separation of the enantiomers and the identification of QE and QA in this work. The stereoselective metabolism of QE in rat in vivo was conducted. The data presented in this study may have some significance for risk assessment.

## Materials and Methods

### 1. Ethics statement

This study and all animal experiments were approved by the local ethics committee (Beijing Association For Laboratory Animal Science), ethical permit number 30749 and carried out with local institutional guidelines.

### 2. Chemicals and Reagents

Rac-quizalofop-ethyl (98%, technical grade) and rac-quizalofop-acid (99%) were obtained from Institute for the Control of Agrichemicals, Ministry of Agriculture of China. Tween 80 and corn oil was obtained from Sigma-Aldrich (St. Louis, MO, USA). Dimethyl sulfoxide, trifluoroacetic acid (TFA), ethyl acetate, n-hexane, acetonitrile, methanol and 2-propanol were purchased from Beijing Chemicals (Beijing, China). Water was purified by Milli-Q water, 18 MΩ·cm. All other chemicals and solvents were of analytical grade and purchased from commercial sources.

### 3. Animal Experiments

Sprague-Dawley male rats weighing 180–220 g were procured from Experimental Animal Research Institute of China Agriculture University and housed in well-ventilated cages with a 12∶12 h light: dark photoperiod. The rats were provided standard pellet diet and water ad libitum throughout the study. The experiments were started only after acclimatization of animals to the laboratory conditions. Before the experiments, the rats were fasted for 12 h, with free access to drinking water at all the times. All the samples were stored immediately at −20°C till the sample processing.

A certain amount of QE dissolved in dimethyl sulfoxide was added in corn oil, after ultrasound and shaking, it turned into a suspension solution and then given to rats by intragastric administration at a dose of 10 mg kg^−1^ b.w. (n = 6) [16]. Blood was sampled from rat tails at 1, 3, 7, 9, 10, 12, 15, 24, 48, 72 and 120 h after the intragastric administration. Control rats received an equal volume of corn oil only. Brain, liver, kidney and lung were collected at 12 h and 120 h respectively. Urine and feces were gathered throughout the study.

The injection solution for intravenous administration was prepared by dissolving QE in tween 80 and adding with sterile saline (5% tween 80, v/v), which was injected into the caudal vein at 10 mg kg^−1^ body weight. Blood was sampled from rat tails at 1/6, 1/2, 1, 2, 3, 5, 8, 12, 24, 48, 72 and 120 h after the intravenous administration.

### 4. Sample Preparations

Kidney, lung, liver, brain and feces were homogenized for 3 min to prepare homogenized tissues. The rat blood (0.2 mL), urine (2 mL) and 0.2 g homogenized tissues were transferred to a 15 mL plastic centrifuge tube with the addition of 5 mL of ethyl acetate. To obtain a better extraction, 100 µL HCl (1 mol L^−1^) was added. The tube was then vortexed for 5 min. After centrifugation at 3500 rpm for 5 min, the upper solution was transformed to a new test tube. Repeat the extraction with another 5 mL of ethyl acetate and combine the upper solution. The extract was dried under a stream of nitrogen gas at 35°C. Then the residue was redissolved in 0.5 mL of 2-propanol or 5 mL of methanol, and finally filtered through a 0.22 µm syringe filter for HPLC and LC -MS/MS analysis.

### 5. Analytical Procedures

QE and QA were analysed by HPLC using Agilent 1200 series equipped with a G1322A degasser, G1311A quatemary pump, a G1329A automatic liquid sampler, G1314B variable wavelength UV detector and Agilent 1200 Chemstation software. A column attemperator (Tianjin Automatic Science Instrument Co. Ltd, China) was used to control column temperature. The chiral column was chiralpak IC (250×4.6 mm, Daicel Chemical Industries, Tokyo, Japan). A 20 µL sample was injected into the column and eluted with a mobile phase of n-hexane: 2-propanol (92: 8 v/v) at a flow-rate of 0.6 mL min^−1^. To get a better separating effect, 0.5% TFA was added to the mobile phase. The temperature of the column was adjusted to 15°C. The elution was monitored by UV absorption at 230 nm and quantification was based on direct comparison of the peak-areas with those of standard. Optical rotatory dispersion (ORD) detector was used to determine the elution orders. LC -MS/MS was used to the identification of QE and QA.

Ultrahigh pressure chromatography was performed using Dionex Ultimate 3000 (Dionex, Sunnyvale, CA, USA) with Hypersil GOLD C_18_ column (2.1×100 mm, 3 µm) at 20°C. The mobile phase was methanol-water-formic acid (70∶30∶0.1%, v/v/v) at a constant flow rate of 0.3 mL/min and the injection volume was 1 µL. A Thermo TSQ Quantum Access Max (Thermo Fisher Scientific, Waltham, MA, USA) with a heated electrospray ionization source (Thermo Fisher Scientific, Waltham, MA, USA) was used to quantitative analysis. MS/MS was operated under the following parameters: spray voltage, 2500 V; vaporizer temperature, 200°C; capillary temperature, 270°C; sheath gas pressure, 30 arb; aux gas pressure, 15 arb. Identification was performed using selected reaction monitoring (SRM) in positive mode for QE and in negative mode for QA, with a scan time of 0.10 s per transition. Data were acquired in SRM mode as summarized in Table 1.

**Table 1:** LC-MS/MS conditions: channel mass, apply mode, retention time.

Analyte	Channel mass	mode	RT(min)
**QE standard**	373+299+271	Positive	2.78
**QA standard**	343+271+243	Negative	1.69
**Blood**	343+271+243	Negative	1.69
**Kidney**	343+271+243	Negative	1.69
**Lung**	343+271+243	Negative	1.69
**Liver**	343+271+243	Negative	1.69
**Brain**	343+271+243	Negative	1.69
**Urine**	343+271+243	Negative	1.69
**Feces**	343+271+243	Negative	1.69

### 6. Method validation

Blank tissues obtained from untreated rats were spiked with rac-QE and rac-QA working standard solutions to generate calibration samples ranging from 0.3 to 60 mg L^−1^. Calibration curves were generated by plotting peak area of each enantiomer versus the concentration of the enantiomer in the spiked samples. The standard deviation (SD) and the relative standard deviation (RSD = SD/mean) were calculated over the entire calibration range. The recoveries were estimated by the peak area ratio of the extracted analytes with an equivalent amount of the standard solution in pure solvents. The limit of detection (LOD) for each enantiomer was considered to be the concentration that produced a signal-to-noise (S/N) ratio of 3. The limit of quantification (LOQ) was defined as the lowest concentration in the calibration curve with acceptable precision and accuracy.

### 7. Data Analysis

Enantiomeric fraction (EF) was used to present the enantioselectivity, defined as: peak areas of (+)/[(+)+(−)]. An EF = 0.5 indicates a racemic mixture, whereas preferential degradation of one of the enantiomers made EF under or over 0.5.

The direct excretion rate (ER) of urine and feces was defined as the following exponential: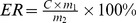.

Where C is the concentration of QA in urine or feces, mg kg^−1^; m_1_ is the amount of urine or feces, g; m_2_ stand for the administered dose of QE. This equation could only reflect the excretion rate approximately base on the assumption that all the QE was metabolized to QA quickly according to the results of this work and the previous studies. The pharmacokinetic parameters such as volume of distribution (Vd) and clearance rate (CL) were generated. The oral bioavailability was calculated as (AUC_oral_/AUC_i.v._)×(dose_i.v_./dose_oral_). The area under the concentration-time curve (AUC) was determined to the last quantifiable concentration using the linear trapezoidal rule and extrapolated to infinity using the terminal phase rate constant. An analysis of variance (ANOVA) was used to determine the statistical differences and p<0.05 was considered to be of statistical significance. Data were presented as the mean ± SD of six parallel experiments.

## Results and Discussion

### 1. Assay Validation

The chromatograms of the control and spiked samples and mass spectrums were shown in Fig. S1 and Fig. S2 in File S1. No endogenous peaks from samples were found to interfere with the elution of QE and QA. The elution sequence of QE and QA was both (+)/(−).

Linearities of all the tissues were shown in Table 2. Over the concentration range of 0.3–60 mg kg^−1^, correlation coefficients (R^2^) were all higher than 0.994. As shown in Table 3 and Table 4, Extraction efficiency of (+)-QE, (−)-QE, (+)-QA and (−)-QA in samples at the concentrations of 0.3, 6 and 60 mg kg^−1^ (n = 3), ranging from 77% to 108% with RSD of 3%–10%. The LOD and LOQ were 0.1 and 0.3 mg kg^−1^, respectively.

**Table 2:** Calibration data of QE and QA enantiomers in different sample matrixes.

Enantiomers	Matrix	Calibration range (mg kg^−1^)	Standard calibration curve		
			Slope	Intercept	R^2^
**(+)-QE**	blood	0.3–60	63.86	−15.13	0.998
	kidney	0.3–60	72.65	59.04	0.998
	lung	0.3–60	71.55	31.69	0.997
	liver	0.3–60	72.19	29.58	0.998
	brain	0.3–60	64.27	1.39	0.996
	urine	0.3–60	68.44	14.52	0.997
	feces	0.3–60	65.66	21.42	0.998
**(−)-QE**	blood	0.3–60	61.38	−16.18	0.997
	kidney	0.3–60	70.41	1.17	0.998
	lung	0.3–60	70.78	44.69	0.995
	liver	0.3–60	68.65	−32.81	0.997
	brain	0.3–60	72.32	17.79	0.996
	urine	0.3–60	63.48	14.7	0.998
	feces	0.3–60	66.05	14.56	0.998
**(+)-QA**	blood	0.3–60	128.15	−43.62	0.998
	kidney	0.3–60	130.19	−32	0.998
	lung	0.3–60	131.65	−26.97	0.995
	liver	0.3–60	130.23	−51.97	0.997
	brain	0.3–60	129.56	−28.1	0.995
	urine	0.3–60	130.48	−43.62	0.998
	feces	0.3–60	132.89	−16	0.998
**(−)-QA**	blood	0.3–60	119.15	−40.81	0.999
	kidney	0.3–60	128.3	−14.85	0.998
	lung	0.3–60	129.87	−14.99	0.997
	liver	0.3–60	130.33	−43.04	0.996
	brain	0.3–60	128.45	−18.42	0.994
	urine	0.3–60	131.68	−32.61	0.999
	feces	0.3–60	126.73	−19.96	0.998

**Table 3:** Extraction efficiency of (+)-QE, (−)-QE in blood, kidney, lung, liver, brain, urine and feces.

Tissues	Recovery(%)					
	(+)-QE			(−)-QE		
	0.3 mg kg^−1^	6 mg kg^−1^	60 mg kg^−1^	0.3 mg kg^−1^	6 mg kg^−1^	60 mg kg^−1^
**Blood**	93.2±5.4	89.4±4.7	88.5±3.7	85.2±5.5	90.3±3.9	92.1±3.3
**Kidney**	80.1±5.8	78.7±3.8	87.3±4.2	78.0±7.9	80.8±6.3	86.7±4.8
**Lung**	86.1±9.7	81.1±6.4	85.2±7.8	88.5±4.4	78.3±4.8	84.1±5.1
**Liver**	86.5±5.5	84.9±7.4	83.9±7.7	80.0±6.9	85.3±4.6	82.7±3.7
**Brain**	77.6±8.8	80.3±9.4	85.2±7.4	84.2±9.3	79.0±6.2	88.6±6.5
**Urine**	90.6±4.4	85.4±5.3	88.9±5.2	101.2±4.8	88.6±4.7	90.1±5.5
**Feces**	86.4±8.4	84.6±8.6	80.9±7.1	78.6±6.5	90.2±5.8	88.5±4.4

### 2. Degradation Kinetics in Rat in vivo

**Figure 2:**
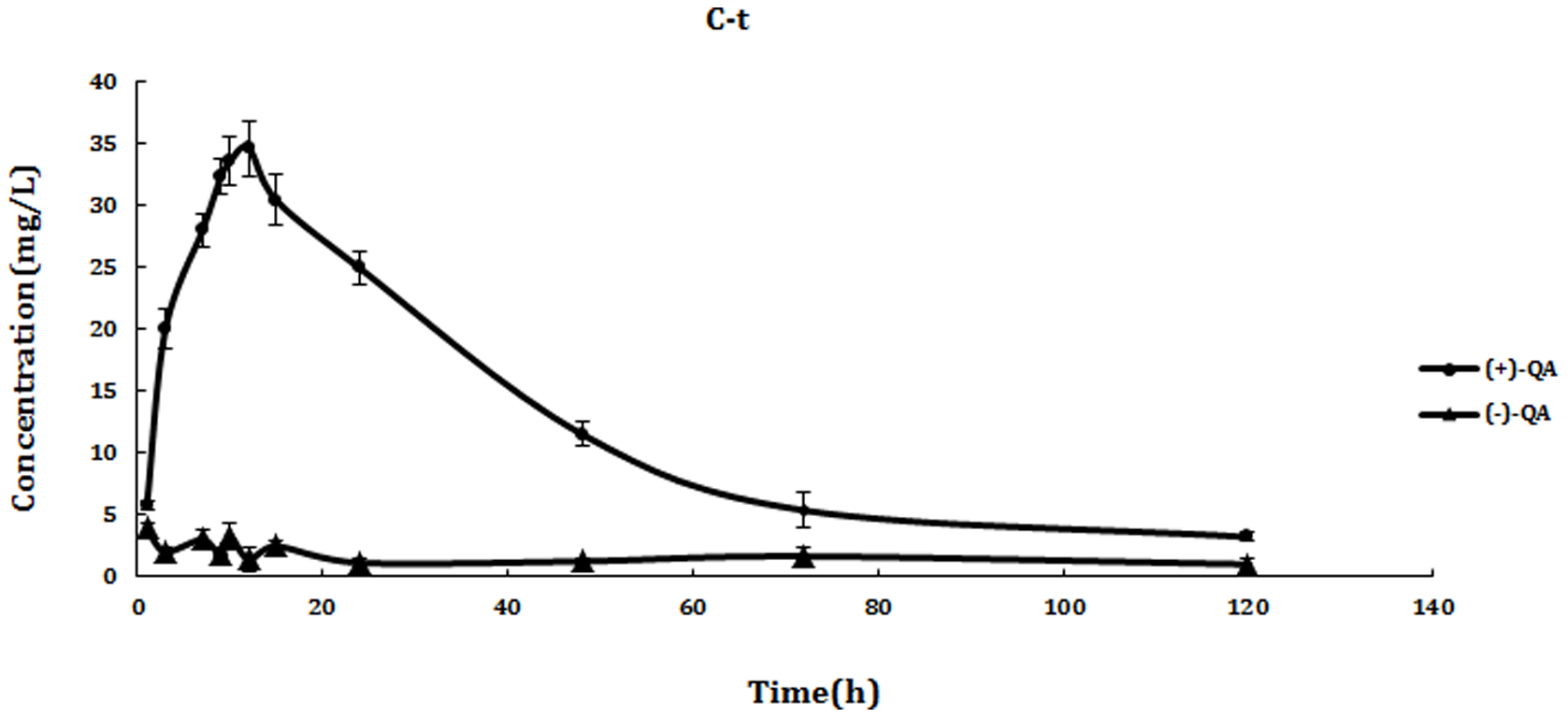
The concentration-time curves of QA enantiomers in blood after intragastric administration. Each point represents the mean ± SD (n = 6). Blood QE level was not detected through the whole study.

**Figure 3:**
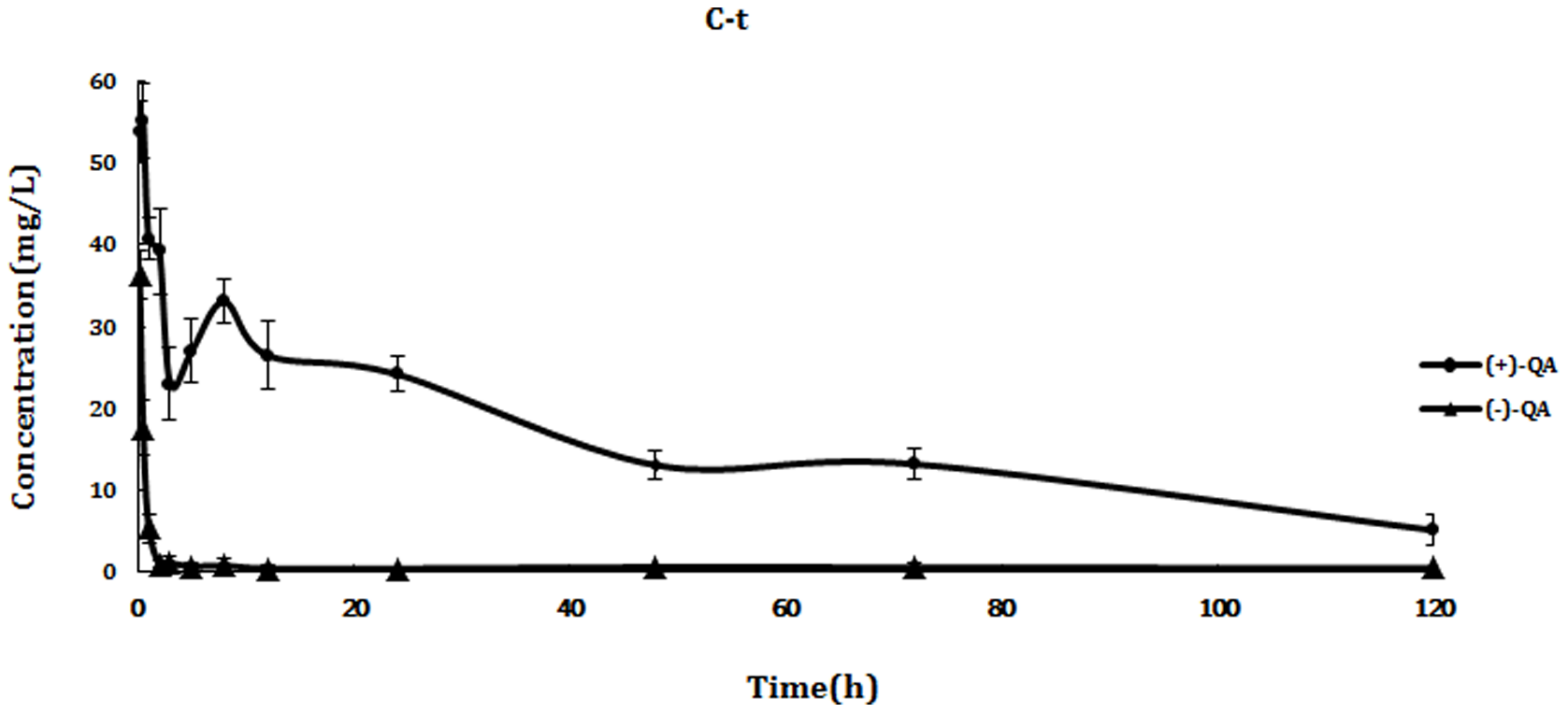
The concentration-time curves of QA enantiomers in blood after intravenous administration. Each point represents the mean ± SD (n = 6). Blood QE level was not detected through the whole study.

**Figure 4:**
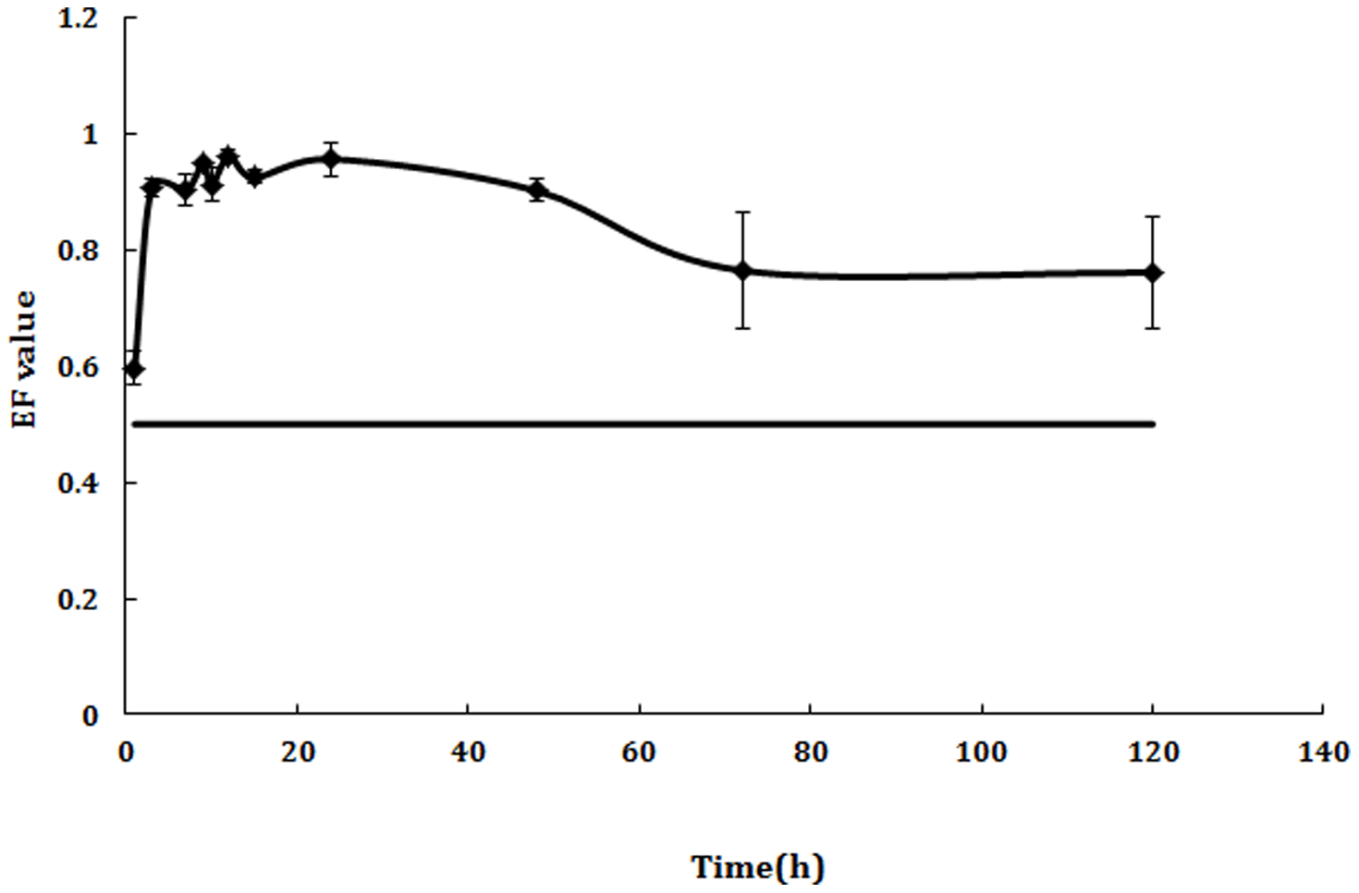
EF-time curve of QA in blood after intragastric administration.

**Figure 5:**
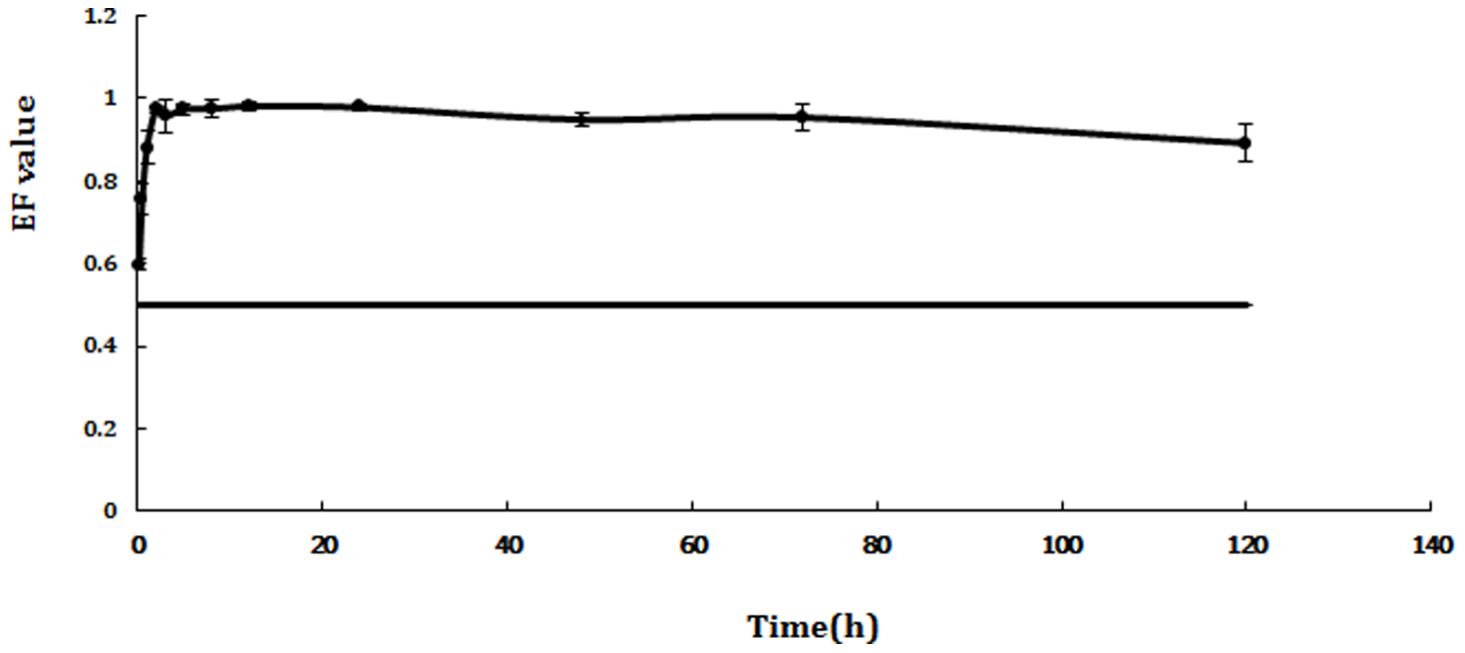
EF-time curve of QA in blood after intravenous administration.

As shown in Fig. 2 and Fig. 3, QE could not be detected in blood after intragastric and intravenous administration of rac-QE, which indicted that QE could be metabolized to QA quickly. However, QA could still be detected even at 120 h in all samples that meant QA could not be easily metabolized by animals. Great difference between the two enantiomers of QA was found in all samples (Fig. 4, Fig. 5). The maximum concentration (Cmax) of (+)-QA in blood was almost ten times higher than that of (−)-QA. Pharmacokinetic parameters and bioavailability of QA after intravenous and oral administration were shown in Table 5. The AUC of (+)-QA and (−)-QA were 1631.202±241.038 mg/L/h and 246.571±70.677 mg/L/h after intragastric administration, and 2239.105±300.554 mg/L/h and 294.751±85.377 mg/L/h after intravenous administration. The oral bioavailability of (+)-QA and (−)-QA were 72.8% and 83.6%. The results revealed a slow clearance of QA from blood.

**Table 4:** Extraction efficiency of (+)-QA, (−)-QA in blood, kidney, lung, liver, brain, urine and feces.

Tissues	Recovery(%)					
	(+)-QA			(−)-QA		
	0.3 mg kg^−1^	6 mg kg^−1^	60 mg kg^−1^	0.3 mg kg^−1^	6 mg kg^−1^	60 mg kg^−1^
**Blood**	101.7±5.1	101.1±3.8	107.7±3.3	102.8±4.3	103.4±4.1	102.4±4.4
**Kidney**	105.2±6.2	104.0±6.5	103.6±5.5	103.5±9.1	101.0±5.2	102.5±5.6
**Lung**	104.2±9.3	107.5±5.5	101.9±6.2	106.5±8.2	105.7±4.6	103.5±6.4
**Liver**	103.5±5.1	105.4±4.3	103.6±5.2	104.4±6.1	103.8±5.4	102.8±7.1
**Brain**	105.2±8.0	101.5±6.6	100.3±8.4	100.6±10.2	106.1±4.9	103.2±5.8
**Urine**	98.9±5.7	96.0±4.4	96.3±5.2	100.3±4.8	101.2±4.8	98.6±6.4
**Feces**	102.6±6.3	100.6±3.5	101.1±6.2	99.8±5.6	101.6±5.3	98.6±8.1

The reason for not detecting QE in blood after intragastric and intravenous administration could be the rapid deesterification of QE in small intestine and blood. The selective uptake, transport across tissues or protein and elimination of enantiomers may be responsible for the enrichment of (+)-QA [17], [18]. The high index of AUC in both intragastric and intravenous administration means that QA was slowly eliminated from plasma and tissues, which may have chronic effects such as reproductive toxicity on rats [15].

**Figure 6:**
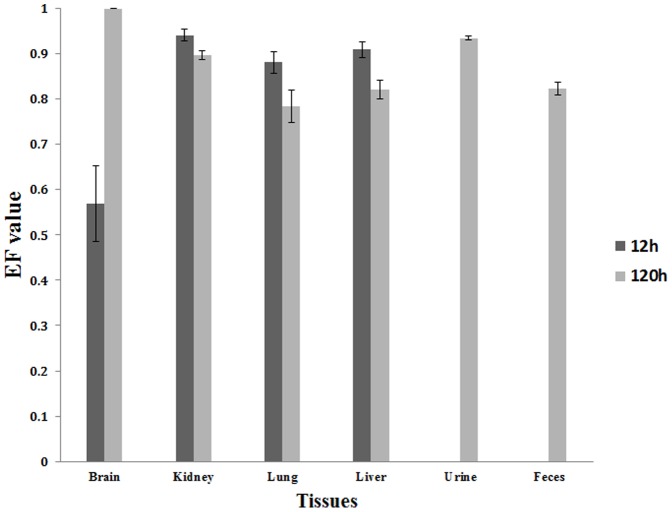
The EF value in brain, kidney, lung, liver, urine and feces.

QE was also not detected in the tissues. The data of the residue of QA at 12 and 120 h in tissues were shown in Table 6. The EF values in brain, kidney, lung, liver, urine and feces were shown in Fig. 6. Both enantiomers could be detected in brain, kidney, lung and liver at 12 h and 120 h except (−)-QA in brain at 120 h. The concentrations of QA in the tissues were in the order of liver>kidney>lung>brain at 12 h and kidney>liver>lung>brain at 120 h. The relative low concentration of (+) and (−)-QA in brain was mainly due to the function of blood-brain barrier [19]. QA was also found in urine and feces. As shown in Table 7, the rats excreted approximately 8.77% and 2.16% of the administered dose by urine and feces based on the calculation. The relative low amount of QA in urine and feces might be attributed to the fact that QA was degraded to further metabolites or QA was transferred to others tissues.

**Table 5:** Pharmacokinetic parameters and bioavailability of QA after intravenous and oral administration (n = 6).

Administration routes	Intravenous administration		Oral administration	
	(+)-QA	(−)-QA	(+)-QA	(−)-QA
**Vd (ml/kg)**	0.279±0.035	9.264±2.519	0.289±0.02	3.302±0.591
**CL (ml/min/kg)**	0.109±0.014	1.069±0.349	0.147±0.005	0.742±0.271
**AUC (mg/h/L)**	2239.105±300.554	294.751±85.377	1631.202±241.038	246.571±70.677
**Bioavailability (%)**	100	100	72.8	83.6

**Table 6:** The concentrations of QA in brains, kidneys, lungs and liver at 12

Tissues	C(mg kg^−1^)/12 h		C(mg kg^−1^)/120 h	
	(+)-QA	(−)-QA	(+)-QA	(−)-QA
**Brain**	1.48±0.23	1.12±0.30	0.93±0.88	nd
**Kidney**	21.77±1.39	1.35±0.25	5.29±0.15	0.60±0.06
**Lung**	15.19±1.20	2.05±0.38	1.65±0.109	0.45±0.07
**Liver**	25.58±1.28	2.53±0.62	4.54±0.23	0.98±0.14

**Table 7:** Excretion rate of (+)-QA (ER_1_) and (−)-QA (ER_2_) by urine and feces.

Excreta	ER_1_	ER_2_
**Urine**	8.20%±0.72%	0.57%±0.04%
**Feces**	1.78%±0.05%	0.38%±0.03%

## Conclusions

The stereoselective metabolism of QE and its primary metabolite QA in rats was conducted. QE was rapidly hydrolyzed to QA and could not be detected in all samples. However, QA still could be detected even at 120 h. High index of AUC indicated that QA was more likely to have chronic toxicity to animal and human, especially to the tissues that contained high concentration of QA, such as liver and kidney. (+)-QA occupy a higher proportion than the (−)-isomer in residues and the faster degradation of (−)-QA might contribute to the enantioselectivity. It was also found that urine excretion was not the main pathway of QA by rat. The data was helpful for full risk assessment of chiral pesticides.

## Supporting Information

File S1 **Figure S1, Representative HPLC chromatograms of QE and QA extracted from untreated and spiked samples.** A1-G1 and A2-G2 represent chromatograms extracted from rat blood, urine, feces, liver, brain, kidney and lung (untreated and spiked with 10 mg L^−1^ of rac-QE and rac-QA respectively). H represents the standard of 10 mg L^−1^ of QA and QE. **Figure S2, Representative MS spectra of QE and QA extracted from untreated and spiked samples.** (A) rat blood; (B) rat urine; (C) rat feces; (D) rat liver; (E) rat brain; (F) rat kidney; (G) rat lung; (1) untreated sample; (2) sample spiked with 1 mg L^−1^ of QE; (3) untreated sample; (4) sample spiked with 1 mg L^−1^ of QA; (H1) standard of 1 mg L^−1^ of QE; (H1) standard of 1 mg L^−1^ of QA.
(DOCX)
