# Supplementary material for: Enantioselective Metabolism of Quizalofop-Ethyl in Rat
Source: PLoS One. 2014 Jun 25;9(6):e101052. doi: 10.1371/journal.pone.0101052 (PMC4071037; doi:10.1371/journal.pone.0101052)
Supplement: File S1 — Figure S1, Representative HPLC chromatograms of QE and QA extracted from untreated and spiked samples. A1-G1 and A2-G2 represent chromatograms extracted from rat blood, urine, feces, liver, brain, kidney and lung (untreated and spiked with 10 mg L−1 of rac-QE and rac-QA respectively). H represents the standard of 10 mg L−1 of QA and QE. Figure S2, Representative MS spectra of QE and QA extracted from untreated and spiked samples. (A) rat blood; (B) rat urine; (C) rat feces; (D) rat liver; (E) rat brain; (F) rat kidney; (G) rat lung; (1) untreated sample; (2) sample spiked with 1 mg L−1 of QE; (3) untreated sample; (4) sample spiked with 1 mg L−1 of QA; (H1) standard of 1 mg L−1 of QE; (H1) standard of 1 mg L−1 of QA. (DOCX) [file pone.0101052.s001.docx]

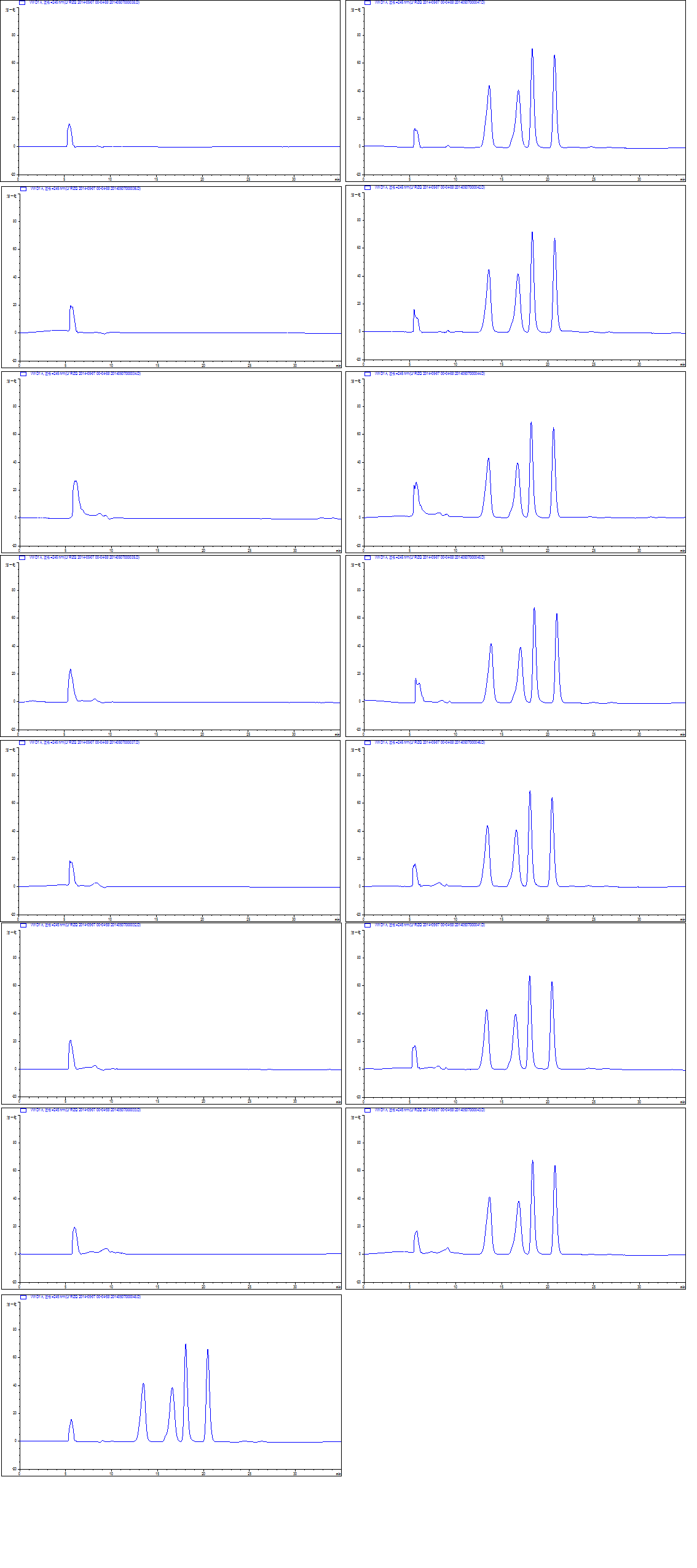


**H**

**G1**

**G2**

**F2**

**F1**

**E1**

**E2**

**D2**

**D1**

**C1**

**C2**

**B2**

**A2**

**B1**

**A1**

**Fig. S1**. Representative HPLC chromatograms of QE and QA extracted from untreated and spiked samples. A1-G1 and A2-G2 represent chromatograms extracted from rat blood, urine, feces, liver, brain, kidney and lung (untreated and spiked with 10 mg/L of rac-QE and rac-QA respectively). H represents the standard of 10 mg/L of QA and QE.

**G3**

**G2**


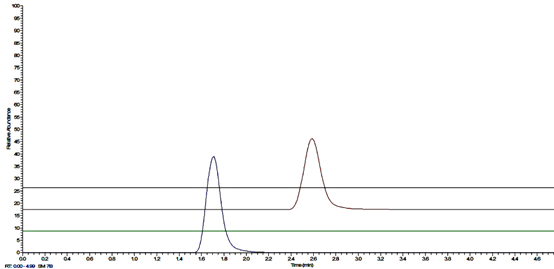

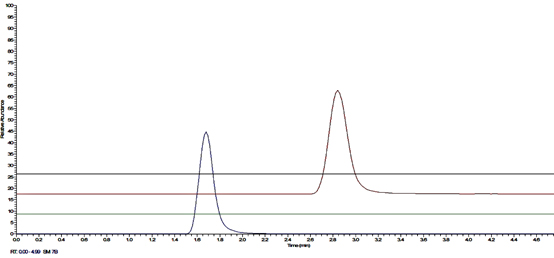

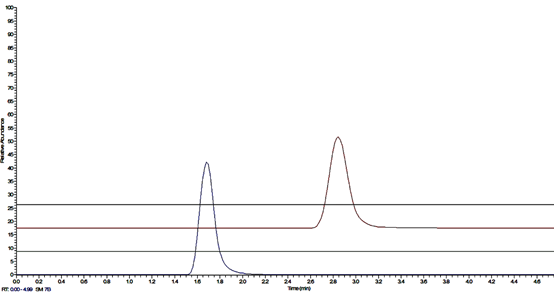

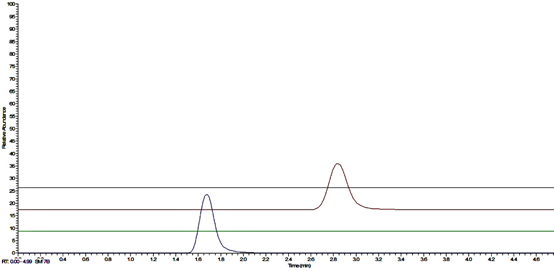

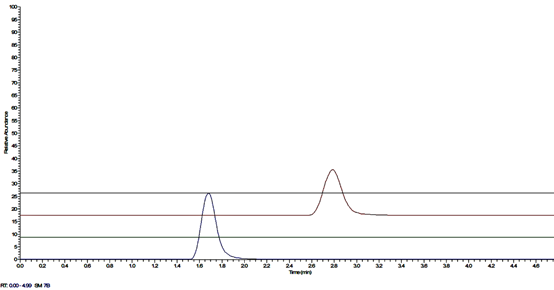

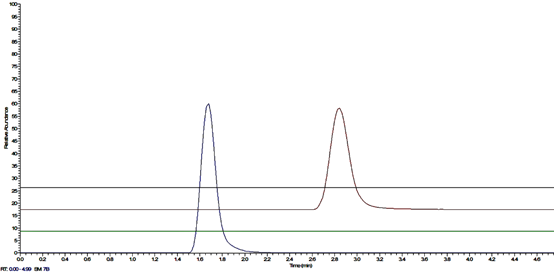

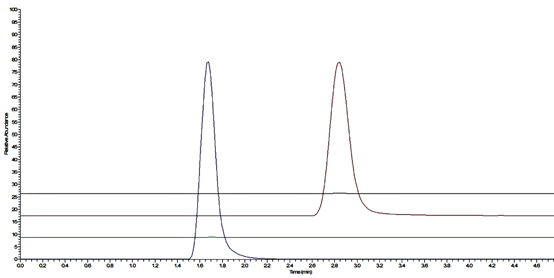

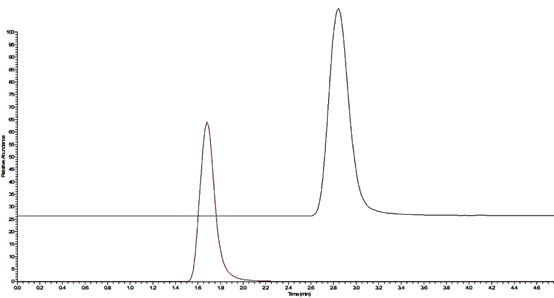


**4**

**3**

**2**

**A**

**1**

**4**

**3**

**1**

**2**

**B**

**C**

**2**

**1**

**3**

**4**

**D**

**2**

**1**

**3**

**4**

**4**

**3**

**1**

**2**

**E**

**4**

**3**

**1**

**2**

**F**

**4**

**3**

**1**

**2**

**G**

**Fig. S2**. Representative MS spectra of QE and QA extracted from (A) rat blood; (B) rat urine; (C) rat feces; (D) rat liver; (E) rat brain; (F) rat kidney; (G) rat lung; (1) untreated sample; (2) sample spiked with 1 mg/L of QE; (3) untreated sample; (4) sample spiked with 1 mg/L of QA; (H1) standard of 1 mg/L of QE; (H1) standard of 1 mg/L of QA.

**H2**

**H1**
